# A fitness index for transplantation of machine-perfused cadaveric rat livers

**DOI:** 10.1186/1756-0500-5-325

**Published:** 2012-06-25

**Authors:** Sinem Perk, Maria-Louisa Izamis, Herman Tolboom, Basak Uygun, Martin L Yarmush, Korkut Uygun

**Affiliations:** 1 Center for Engineering in Medicine, Massachusetts General Hospital, Harvard Medical School, and the Shriners Hospitals for Children, 51 Blossom Street, Boston, MA, 02114, USA; 2 Division of Cardiac and Vascular Surgery, University Hospital Zurich, Zurich, Switzerland; 3 Department of Biomedical Engineering, Rutgers University, Piscataway, NJ, USA

**Keywords:** Transplantation index, Principal component analysis (PCA), Partial least squares (PLS), Extracorporeal liver perfusion, Donors after cardiac death

## Abstract

**Background:**

The 110,000 patients currently on the transplant waiting list reflect the critical shortage of viable donor organs. However, a large pool of unused organs, from donors after cardiac death (DCD) that are disqualified because of extensive ischemic injury, may prove transplantable after machine perfusion treatment, fundamentally impacting the availability of treatment for end-stage organ failure. Machine perfusion is an ex-vivo organ preservation and treatment procedure that has the capacity to quantitatively evaluate and resuscitate cadaveric organs for transplantation.

**Methods:**

To diagnose whether an organ was fresh or ischemic, an initial assessment of liver quality was conducted via dynamic discriminant analysis. Subsequently, to determine whether the organs were sufficiently viable for successful implantation, fitness indices for transplantation were calculated based on squared prediction errors (SPE) for fresh and ischemic livers.

**Results:**

With just three perfusate metabolites, glucose, urea and lactate, the developed MPLSDA model distinguished livers as fresh or ischemic with 90% specificity. The SPE analyses revealed that fresh livers with SPE_F_ < 10.03 and WI livers with SPE_WI_ < 3.92 yield successful transplantation with 95% specificity.

**Conclusions:**

The statistical methods used here can discriminate between fresh and ischemic livers based on simple metabolic indicators measured during perfusion. The result is a predictive fitness index for transplantation of rat livers procured after cardiac death. The translational implications of this study are that any donor organ procured from controlled, but most especially from uncontrolled cardiac death donors, will be objectively assessed and its recovery monitored over time, minimizing the critical loss of otherwise viable organs.

## Background

The major untapped pool of donor organs that could be used to alleviate the critical shortage of transplantations derives from donors who have experienced cardiac death [1,2]. Approximately 4,000 patients perish while waiting for a donor liver every year [3], while the estimated pool of livers from donors after cardiac death (DCD) with recoverable ischemic times of 30 – 60 minutes is on the order of 6,000 grafts per year [2].

In the absence of cardiac output, ischemic damage increases in severity as a function of time. Beyond a certain cutoff (about 30 minutes for the liver) graft survival in the recipient falls dramatically [4]. Preclinical studies with extracorporeal machine perfusion systems in porcine and murine models of DCD livers [5-11], including from our group [12-14], indicate that up to 60 minutes of warm ischemic damage can be successfully reversed, whereas static cold storage in preservation medium, the current clinical gold standard, simply exacerbates the damage and recipient animals do not survive. Research in machine perfusion systems is subsequently a very active field in donor organ recovery and preservation [15-18].

In humans, cardiac death frequently occurs in uncontrolled environments (uDCD). Hence without objective metrics of ischemic duration and organ viability, uDCD organs cannot be safely transplanted. A suitable framework for developing a fitness index that describes whether an organ is transplantable or not could be based on the dynamic correlation between metabolites measured during perfusion and evaluated with multivariate statistical process monitoring (SPM) [19,20]. Normothermic (37 °C) Machine Perfusion (NMP) is especially amenable to this analysis since it allows measurable metabolic activity to occur in the organ.

The aim of this work is the development of a fitness index for transplantation for DCD livers based on the organ ischemic injury level and metabolite transient profiles during perfusion, as a proof-of-concept in a rat model. To create such an index, we compared NMP treatment of fresh, 60 min, and 90 min warm ischemic (WI) livers, where only the last group did not survive transplantation. We first constructed a multi-way partial least squares discriminant analysis (MPLSDA) model to assess what the acceptable degree of ischemic damage of each liver was for successful transplantation (> 1 month survival) and classified them as fresh or ischemic based on their urea, lactate and glucose profiles during perfusion. We also constructed fresh liver and ischemic liver successful perfusion models using multi-way principal component analysis (MPCA). Once a new liver was identified as fresh or ischemic, the corresponding MPCA model determined the SPE-distance of the given liver to the reference livers used in model-building, hence, constructing a predictive fitness index for transplantation based on SPE statistics. If the fitness index for the new liver fell within the 99% confidence limits, this would indicate that the liver is suitable for transplantation.

## Methods

### Animals

Male Lewis Rats weighing 200-300 g were obtained from Charles River Laboratories (Wilmington, MA) and maintained in accordance with National Research Council guidelines. The Subcommittee on Research Animal Care, Committee on Research at the Massachusetts General Hospital, approved the experimental protocols. All animals were allowed to acclimatize for at least 2 days prior to any experimentation. Full details of the liver isolation protocol for donor livers are explained in [14]. WI was induced by placing livers in a temperature-controlled chamber filled with saline and maintained at 34 ± 0.1 °C during which time the portal vein and vena cava were cuffed. Ex vivo ischemia ensured a constant temperature [21] and enabled a severe model of warm ischemia [8]. Livers that have been exposed to 60 minute or 90 minute warm ischemia were flushed with saline and then connected to the perfusion system. Fresh livers were flushed through the PV with 10mls of saline upon clamping the vein in situ and were then placed in a bowl of room temperature saline to be cuffed at the PV and IVC; average warm ischemic time prior to reperfusion was 10 ± 2 minutes. Procured livers were grouped in three with respect to the duration of warm ischemia they were exposed to: 1) Fresh (F; n = 10), 2) 60 min warm ischemia (WI; n = 6), and 3) 90 min warm ischemia (WI90; n = 3).

### Normothermic liver perfusion and transplantation

The perfusion medium contained phenol red-free Williams Medium E (WE, Sigma Chemical, St. Louis, MO). WE was supplemented with 2 u/L insulin (28.85 units/mg Humulin, Eli Lily, Indianapolis, IN), 100,000 u/L penicillin, 100 mg/L streptomycin sulfate (Gibco, Invitrogen, Grand Island, NY), 0.292 g/L L-glutamine (Gibco), 10 mg/L hydrocortisone (Solu-Cortef, Pharmacia & Upjohn, Kalamazoo, MI), and 1000 u/L heparin (APP, Schaumburg, IL). The primary circuit of the perfusion system comprised perfusion medium (perfusate) that recirculated by means of a peristaltic pump through a jacketed perfusion chamber, a membrane oxygenator, a heat exchanger, and a bubble trap. The oxygenator was gassed with a mixture of 74%N_2_/21%O_2_/5%CO_2_ and 100% O_2_ to maintain a constant pH. Fresh rat plasma (25% v/v) and erythrocytes (18-20% v/v) were collected earlier and added to the perfusate. The total perfusate volume was 55 to 60 mL. Perfusate hematocrit was sustained, nutrients were replenished, and metabolism by-products were diluted through dialysis. A hollow fiber dialyzer with a 2200 cm^2^ membrane area and a 30 kDa nominal molecular cutoff weight (SpectrumLabs, Rancho Dominguez, CA) enabled counter-current mixing of perfusate in the primary circuit with a reservoir of WE (dialysate) in a secondary circuit. Temperature within the system was maintained at 37 °C.

Upon completion of cuffing of fresh livers (~ 5 min) and after the period of warm ischemia for WI livers, they were immersed in perfusate in the perfusion chamber. Livers were perfused at a constant flow rate through the portal vein while maintaining portal pressure between 10 and 12cmH_2_O. The effluent flowed freely from the SHVC and IVC into the surrounding medium. When the recipient hepatectomy was prepared, the liver was disconnected from the circuit, rinsed in a bowl of saline at room temperature, and weighed again before transplantation. A modification of the cuff technique designed by Kamada and Calne [22-24] was used for orthotopic rat liver transplantation. The anhepatic phase of the procedure was typically 13-15 minutes and did not exceed 17 minutes. Animals were hydrated with 8 ml/kg of warm (37°C) lactated Ringer’s solution with 5% dextrose and 2 ml/kg of NaHCO_3_ 7%w/v (Abbott, North Chicago, IL) by penile vein injection. Following transplantation, the animals were allowed to recover from anesthesia in separate cages under an infrared lamp for half an hour, and subsequently returned to regular housing. During the first 12 hours post-transplantation animals were checked every 2 hours and subsequently every 8 hours for one week, and daily afterwards.

### Metabolite sampling

Perfusate and dialysate samples (1 mL) were collected hourly from the liver effluent and reservoir, respectively. Perfusate samples were first spun down at 3000 g before storing the supernatant at -80°C. We focused on readily-measured metabolites: Urea was assayed by reaction with diacetyl monoxime using a commercial assay kit (BUN, Sigma-Aldrich, St. Louis, MO). Lactate was measured using the enzymatic conversion to pyruvate and hydrogen peroxide with lactate oxidase from a commercially available kit (Trinity Biotech, Berkeley Heights, NJ). Glucose measurements were quantified with an enzymatic assay kit through conversion to 6-phospo-gluconate (Glucose assay kit, Sigma). Data consisted of glucose, lactate, and urea measured hourly for each perfusion. 10 fresh livers, 6 (60 min) and 3 (90 min) WI livers were each perfused for 6 hours. Fresh livers and livers that were exposed to 60 minutes of WI were successfully transplanted with >1 month survival (Table1).

**Table 1:** Initial conditions and number of grafts for 6 hours of machine-perfusion

**Initial condition**	**Total number of livers used in analysis**	**Transplant result**
Fresh Livers	10	Survival (10/10)
60 min warm ischemia (WI)	6	Survival (6/6)
90 min WI	3	Failure (3/3)

### Statistical analysis

Statistical process control methodologies for batch processes were used in the analyses. Each perfusion is a batch process with a finite duration. The process data is a 3D array that consists of (perfusions x number of variables x time). An (I x J x K) data array can be unfolded by preserving the batch direction I and augmenting the J variable measurements taken at each time point *k* (k = 1,…, K) side by side resulting in an I x JK matrix [19,20,25,26]. This unfolding direction is the best to use in the SPM of batch processes since it considers the batch-to-batch variations. The (I x JK) unfolding of the batch data yields datasets which consist of few observations and many variables. Unlike ANOVA and classical methods of statistics, the multivariate projection methods such as MPCA, MPLSDA work effectively on these short and wide datasets with constrained sample sizes [19]. 10 fresh liver perfusions and 6 WI liver perfusions were statistically sufficient for modeling using SPM methodologies. An additional three replicates were performed with 90 minute WI liver perfusions for testing. Please note that unlike ANOVA designs, equal replicates in all groups is not necessary. All variables (i.e. the metabolite concentrations) were mean-centered and unit-variance scaled for further statistical analyses.

### Multiway partial least square discriminant analysis (MPLSDA)

All Fresh and 60 min WI liver perfusions were transplanted successfully. However, during perfusion 60 min warm ischemic livers displayed significantly different metabolic profiles compared to fresh livers hence, indicating the need for the fitness for transplantation to be evaluated separately for reversibly ischemic and fresh livers. To achieve this, first the quality of the liver had to be determined as fresh or 60 min WI.

To be able to classify livers as ischemic or fresh, an algorithm that can discriminate different metabolic states from the dynamic metabolite data collected during successful perfusions is required. Partial least squares or projections to latent structures (PLS) is a regression technique that is used to connect the information in two blocks of variables, namely the predictor block **X** and response block **Y**. An algorithm for Nonlinear Iterative Partial Least Squares (NIPALS) is provided in [27]. PLS is extended to multi-way PLS (MPLS) for three dimensional (3D) data as (perfusions x variables x time). For new data coming from a new perfusion, **x**_new_, MPLS computes the response variables **y**_new_ using the model regression coefficients.

MPLS discriminant analysis (MPLSDA) is used to classify observations or independent experiments in **X** as belonging to one of several a-priori known classes in **Y** based on their relationship [19]. The predictive power of MPLSDA was evaluated by case-re-sampling cross validation technique [28,29]. In this approach, the data set was sampled randomly multiple times to create training and validation data sets. For each sampling, five samples from the fresh liver group and five samples from the 60 min WI liver group were selected randomly and the remaining samples were used for testing the model. The maximum selection threshold, which is the maximum number of times same perfusion can be used in the same model, was set to 2. The selection threshold was added to avoid the same samples being selected most of the time.

For the analysis of fresh and ischemic livers, **X** consists of the three metabolites’ six hour trajectories for ten selected perfusions: five 60 minute WI and five fresh; and **Y** consists of two columns representing the class membership of each of the ten batches. For a fresh liver perfusion batch (class 1), the corresponding row of **Y** is [1, 0], whereas, for a WI liver batch the row is set to [0, 1]. When the new data **x**_new_ is projected onto the MPLSDA model, $\hat{y}$ vector is calculated. The predicted class is the numerical maximum of the normalized $\hat{y}$ vector.

### Multi-way principal component analysis (MPCA)

For the statistical modeling of liver perfusions with MPCA, two MPCA models were built to capture the metabolic functioning of fresh and warm ischemic (60 minute) livers during perfusions.

PCA captures the correlation structure between the variables in **X** and forms a model plane with fewer dimensions (R principal component (PC) directions) using only the R largest variance directions [30]. R is chosen such that adding additional components to the model does not provide additional significant information. Instead of working with highly correlated collinear variables (**X**), PCA yields fewer and uncorrelated projections (scores (**T**)).

(1) $$X=TP^{T}+E=\hat{X}+E\;\text{;}\;\hat{t}=x_{\mathrm{new}}P\;\text{;}\;e_{\mathrm{new}}=x_{\mathrm{new}}-\hat{t}P^{T}$$

PCA is performed by singular value decomposition in the covariance of **X** and the loadings (eigenvectors) **P** are derived. The R eigenvectors are the R highest variance directions. Scores (**T)** are the new uncorrelated variable projections onto the newly derived PCA plane. **E** is the residual matrix. Score bi-plots can then be used to reveal the degree of similarity between independent samples, possible clustering among the samples, and any outliers. Similar to MPLS, PCA has been extended to MPCA for 3D arrays [20,25,31] to handle the time-series data obtained during perfusion.

### Squared prediction error (SPE)

SPE statistic [20,25,26,31,32] captures the large deviations from a reference set and is calculated for the new batch using the residuals **e**_new_[20],

(2) $$SPE=e_{\mathrm{new}}e_{\mathrm{new}}^{T}=\sum_{c=1}^{KJ}E_{new,c}^{2}$$

A large SPE statistic indicates that the observation under consideration contains a different correlation structure and is not explained by the model. $\;\chi_{2m^{2}/\upsilon ,\alpha }^{2}$ is the critical value of the chi-squared variable with 2 m^2^/υ degrees of freedom at significance level α. m and υ are the sample mean (m) and variance (υ) of the SPE values of the reference batches [25].

For instance, if a given liver perfusion contains a correlation structure that is not captured by the MPCA model, it will be indicated by a large SPE value. As such, SPE effectively distills the dynamic metabolite profiles to a single variable that quantifies the distance of any liver perfusion from a set of reference perfusions. For fresh livers, MPCA model 99% confidence limits will determine the maximum acceptable value of the SPE statistic for a fresh liver perfusion to be deemed fit for transplantation. Similarly, MPCA model of transplantable ischemic livers will determine the upper limit for the SPE statistic for an ischemic liver to be considered transplantable.

## Results

### Degree of ischemia and classification

MPLSDA can classify a perfused graft as healthy or ischemic so that a decision can be made about the organ quality and eventually, on its fitness for transplantation by comparing it to sets of successfully transplanted fresh and ischemic livers.

Five fresh liver and five 60 min WI liver perfusions were used in the MPLSDA model (R = 4), 4 PCs explain more than 86% of the variation in **X** and 99% variation in **Y**.

**Figure 1:**
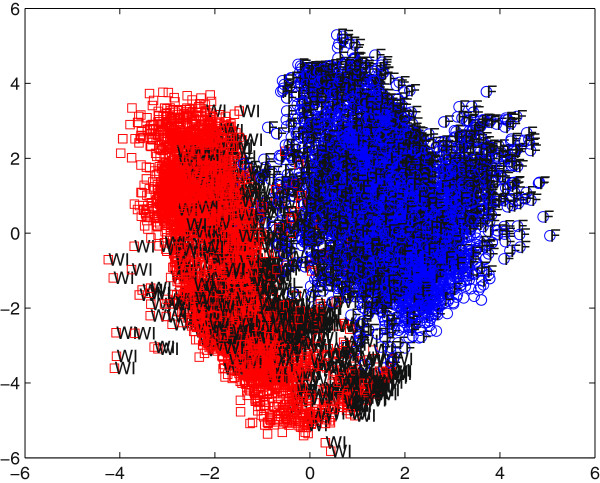
**MPLSDA fresh and 60 minute WI liver clusters.** Red squares and blue circles denote ischemic livers and fresh livers, respectively.

Two distinct clusters were observed in the score plot (Figure1). 60 min ischemic livers and fresh livers have different urea, lactate, and glucose concentration profiles; hence it was evident that the two groups should be modeled separately for the determination of separate fitness indices for transplantation.

### Fitness for transplantation indices for fresh and ischemic livers

Once the MPLSDA determined whether a liver was fresh or ischemic, the corresponding MPCA model and its 99% SPE confidence limits was used as a basis to determine the organ’s fitness for transplantation. Accordingly, we considered SPE statistic as a heuristic index for rats for fitness of transplantation, with 99% confidence values of SPE_F_ < 10.03 and SPE_WI_ < 3.92 indicating fitness.

**Figure 2:**
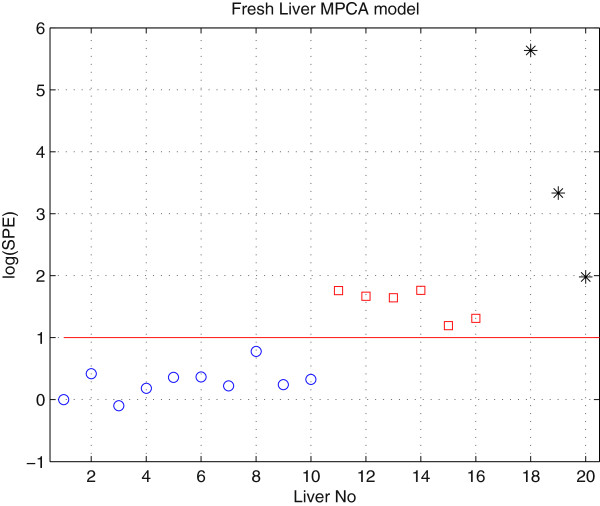
**Log(SPE) distances to the fresh liver MPCA model.** Fresh livers, denoted by blue circles, are within the 99% model confidence limits (SPE_F_ = 10.03). 60 min and 90 min ischemic livers are denoted by red squares and black stars, respectively.

**Figure 3:**
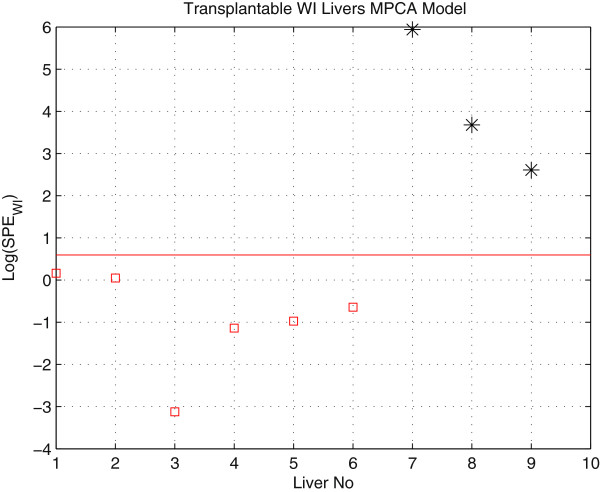
**Log(SPE) distances to the 60 min WI liver MPCA model.** 60 min WI livers, which were successfully transplanted, are denoted by red squares. The 99% model confidence limits (SPE_WI_ = 3.92). 90 min ischemic livers, denoted by black stars, are outside the confidence limits.

The transplantable fresh livers model and the transplantable ischemic livers model are shown in Figures 2 and 3. In the fresh liver MPCA model, the 60 min and the 90 min ischemic livers are outside of the limits since they do not retain the same concentration profiles of transplantable fresh livers (Figure2). The SPE values for 90 min WI livers are the largest, indicating a larger qualitative difference to fresh livers than 60 min WI livers. Next, the 90 min WI liver perfusions were projected onto the 60 min WI liver perfusions MPCA model. The 99% confidence limit, SPE_WI_ < 3.92, for transplantable warm ischemic livers was used and all of the 90 min WI livers were outside the limits, affirming that these livers were not fit for transplantation (Figure3).

The classification followed by SPE calculation scheme for the determination of transplantation fitness yields the results given in Table2. For each liver, the incorrect predictions are summed and averaged over the number of times the liver has been picked for model-testing out of 1000 runs. The incorrect predictions are due to the deficiency of the MPLSDA classification (specificity ~ 90%), where some of the fresh livers are confused as ischemic livers. This scheme is able to successfully predict (no misclassifications) that the 90 min ischemic livers were not fit for transplantation.

**Table 2:** Fitness for transplantation predictions

**Fresh liver No**	Incorrect predictions* (%)	**60 min WI liver No**	Incorrect predictions* (%)	**90 min WI liver No**	Incorrect predictions* (%)
1	10.08	1	0	1	0
2	1.53	2	0	2	0
3	21.35	3	3.60	3	0
4	0	4	0		
5	0	5	17.85		
6	0	6	0		
7	0				
8	3.63				
9	7.82				
10	0				

*Average values for each liver are calculated as the ratio of number of incorrect predictions to the total number of times the liver was picked in 1000 runs.

## Discussions and conclusion

In this work we introduce statistical process monitoring methodologies that employ liver metabolic performance to evaluate the degree of ischemic injury cadaveric organs have sustained and, based on the degree of injury, determine the fitness for transplantation.

The multivariate analyses performed demonstrated that ischemic and fresh rat livers are easily distinguishable from each other and accurately classifiable based on their metabolic function after exposure to different durations of ischemia. Our MPLSDA-based classification algorithm was able to determine the quality of a new liver as fresh or ischemic with ~90% specificity. Once the liver quality was determined, MPCA-based SPE statistic enabled its comparison to reference fresh or reference ischemic livers, gauging the extent of recovery to a transplantable state.

We therefore conclude that the SPE statistic can be employed as an accurate and continuous index of fitness for transplantation for perfused rat livers. Based on our data, cutoff values for SPE for fresh and ischemic livers were determined to be used as heuristic limits of fitness for transplantation. This analysis can be further expanded to explore the correlations between metabolic function and traditional tests of cellular injury, and translated to human livers for a clinically applicable graft quality assessment and an index of transplantation.

The analyses performed in this work all confirm that a metabolic index of ischemic injury is a feasible idea for evaluation of perfused ischemic livers, and such a measure would be of significant use in utilization of DCD livers for transplantation. This study demonstrates the power of SPM methodologies in achieving this goal; however, further work is needed to enrich the data used here for more sophisticated metabolic analyses which may reveal underlying details of cellular injury in the context of transplantability, as well as translation to clinical studies.

## Abbreviations

DBD, Donors after brain death; DCD, Donors after cardiac death; IVC, Inferior vena cava; MPCA, Multi-way principal component analysis; MPLS, Multi-way partial least squares; MPLSDA, Multi-way partial least squares discriminant analysis; NIPALS, Nonlinear iterative partial least squares; NMP, Normothermic machine perfusion; PC, Principal component; PCA, Principal components analysis; PLS, Partial least squares; PV, Portal vein; SPE, Squared Prediction Error; SPM, Statistical Process Monitoring; uDCD, Uncontrolled DCD.

## Authors’ contributions

KU had full access to all of the data in the study and takes responsibility for the integrity of the data and the accuracy of the data analyses. Study concept and design, SP, MLI, BEU, KU Acquisition of data, MLI, HT, BEU. Analysis and interpretation of data, SP, MLI, KU Transplantation studies, HT Drafting of the manuscript, SP, KU Critical revision of the manuscript for important intellectual content, SP, MLI, BEU, MY, KU Statistical analysis, SP, KU Obtained funding, MY, KU Administrative, technical, or material support, MY, and KU. All authors contributed to the preparation of the report. All authors read and approved the final manuscript.
